# External assets and psychosocial adaptation in left-behind children: stress mindset as mediator and environmental sensitivity as moderator

**DOI:** 10.3389/fpsyg.2026.1840516

**Published:** 2026-07-02

**Authors:** Shuhan Lin, Ruijia Fu, Qian Zhong, Yuanlong Jiang, Shunyi Jiang, Yun Tao

**Affiliations:** 1 Faculty of Education, Yunnan Normal University, Kunming, China; 2 Kunming No. 5 Middle School, Kunming, China; 3 School of Psychology, Shaanxi Normal University, Xi’an, China; 4 Mental Health Education and Counseling Center, Kunming University, Kunming, China

**Keywords:** environmental sensitivity, external assets, left-behind children, psychosocial adaptation, stress mindset

## Abstract

**Introduction:**

The psychosocial adaptation of rural left-behind children has become a significant social concern. While previous research has mainly focused on developmental vulnerabilities from a deficit perspective, less attention has been paid to the external resources and positive individual traits that facilitate optimal development. To address this gap, grounded in the positive youth development perspective, this study constructed a moderated mediation model to examine the mediating role of stress mindset in the relationship between external assets and the psychosocial adaptation of left-behind children, as well as the moderating role of environmental sensitivity in this process.

**Methods:**

A questionnaire survey was conducted using a stratified cluster random sampling approach among 2,639 rural children, with 1,590 left-behind children in the target group and 1,049 non-left-behind children in the control group. Data were collected using standardized instruments, including the External Assets Subscale, the Stress Mindset Scale, and the Environmental Sensitivity Scale.

**Results:**

(1) The levels of external assets and psychosocial adaptation in left-behind children were significantly lower than those of their non-left-behind counterparts; (2) external assets were positively associated with psychosocial adaptation among left-behind children, and stress mindset partially mediated this association; (3) Environmental sensitivity moderated the association between stress mindset and psychosocial adaptation; specifically, this association was stronger among children with higher environmental sensitivity.

**Conclusion:**

These findings suggest that external assets are positively associated with psychosocial adaptation via stress mindset, with environmental sensitivity potentially moderating this indirect association. Moreover, the positive association between stress mindset and psychosocial adaptation was stronger among children with higher environmental sensitivity.

## 1 Introduction

Since the late 1970s, China’s rapid urbanization has led to large-scale migration of rural labor to urban areas. Constrained by the dual urban-rural structure and other institutional factors, several migrant workers cannot bring their children with them, leaving them behind in their rural hometowns. This has led to the emergence of a distinct demographic group known as “rural left-behind children.” Rural left-behind children are defined as minors under the age of 16 years who have been left at home by both parents or one parent, while the other lacks guardianship capacity due to parental migration for work (Zhao et al., 2013). According to the Bulletin of China’s Seventh National Population Census, the number of left-behind children has reached 66.93 million, accounting for approximately 22.5% of the nation’s total child population. Owing to the prolonged absence of parents, this vast group generally faces significant challenges of poor psychosocial adaptation, such as loneliness, depression, and externalizing problem behaviors (Xiong et al., 2024; Lin et al., 2025). Psychosocial adaptation refers to the process by which individuals adjust their psychological states and behavioral patterns to meet environmental demands and achieve personal goals during their interaction with the environment (Hou et al., 2022). Psychosocial adaptation is a multidimensional construct, and previous studies have characterized it using indicators such as wellbeing, depression, peer relationships, and externalizing problem behaviors (Lan and Sun, 2022; Hou et al., 2022). Compared to non-left-behind children, left-behind children exhibit lower levels of wellbeing (Fan et al., 2025), prosocial behavior (Li X. et al., 2025), and peer relationships (Dai and Peng, 2021), with higher levels of depression (Fan et al., 2018) and externalizing problem behaviors (Yang and Huang, 2022). However, recent studies have found that a considerable proportion of left-behind children demonstrate good psychosocial adaptation (Chen et al., 2024). This suggests that being “left-behind” is not synonymous with being “problematic,” and the psychosocial adaptation of left-behind children presents significant individual differences during development. Therefore, it is necessary to move beyond the deficit perspective and systematically examine the factors and mechanisms underlying the psychosocial adaptation of left-behind children.

Previous studies have predominantly adopted a deficit perspective to investigate the negative impact of prolonged parental absence on the psychosocial adaptation of left-behind children, providing an important basis for identifying developmental risk factors. However, to a certain extent, the deficit perspective has overlooked the external assets that left-behind children may obtain during their development, as well as their positive traits in coping with environmental challenges. In recent years, with the rise of the Positive Youth Development (PYD) perspective, researchers have increasingly recognized that even in adverse circumstances, left-behind children possess developmental potential. Parental migration for work does not necessarily constitute a risk factor for their positive development (Zhang and Zhang, 2023). Wen et al. examined the impact of parental migration on the development of rural left-behind children in China and found that there were almost no differences in positive youth development across different parental migration statuses (father only, mother only, both parents, and neither parent migrating) (Wen et al., 2015). Therefore, promoting the psychosocial adaptation of left-behind children should not be limited to addressing maladjustment; rather, it should focus on positive developmental factors (Lerner et al., 2015). According to relational developmental systems theory, individual development results from the dynamic interaction between the individual and the external environment (Lerner, 2009). Similarly, the positive youth development perspective posits that positive individual traits and supportive contextual environments jointly facilitate the formation of positive psychological and behavioral outcomes. If children can effectively utilize external assets and develop positive cognition and optimistic coping strategies in response to challenges, they may mitigate the adverse effects of unfavorable circumstances and achieve better psychosocial adaptation (Fan et al., 2023). As individuals differ in the degree to which they are affected by their environment (Belsky and Pluess, 2013), the strength of these effects on psychosocial adaptation may vary across children. Accordingly, this study explores how environmental factors (external assets) and individual factors (stress mindset and environmental sensitivity) are associated with psychosocial adaptation among left-behind children.

### 1.1 External assets and the psychosocial adaptation of left-behind children

External assets originate from the developmental assets framework proposed by Benson et al. and refer to the support and growth opportunities provided to individuals by external contexts, such as families, schools, and communities, including support, empowerment, boundaries and expectations, and the constructive use of time (Benson, 2002; Chang and Zhang, 2013). Within this framework, external assets are regarded as protective factors that promote positive youth development (Martin-Barrado and Gomez-Baya, 2024). For left-behind children who endure long-term parent-child separation, the support and growth opportunities afforded by the external environment may be of paramount importance for the development of their psychosocial adaptation. However, systematic evidence regarding the relationship between external assets and the psychosocial adaptation of left-behind children remains limited in existing literature. Previous studies have mainly focused on single or specific external assets and their associations with certain indicators of psychosocial adaptation. Specifically, within the family domain, left-behind children who maintain a high level of trust in their primary caregivers (including support) may better cope with long-term parent–child separation through caregiver interactions and may partly satisfy their basic psychological needs, which may relate to higher subjective wellbeing (Chai et al., 2019; Bãlţãtescu et al., 2023). In addition, online parent-child communication, as a form of family support (included in support), has been positively associated with the social adaptation of left-behind children (Zhang, 2025). School empowerment (a component of empowerment) has also been positively associated with the mental health of left-behind children (Li J. et al., 2025). In the community context, neighborhood cohesion and trusting relationships with caregivers (including support) are positively correlated with the subjective wellbeing of left-behind children (Chai et al., 2019). Although these studies provide preliminary evidence across ecological levels, they have mainly focused on a single or a limited number of external assets, leaving the comprehensive and integrative associations between these assets and psychosocial adaptation insufficiently examined. According to the accumulation hypothesis of developmental asset theory, accumulating external assets is associated with more positive developmental outcomes among youth (Chang and Zhang, 2013). Based on this hypothesis and prior empirical findings, we hypothesize that higher levels of external assets are associated with better psychosocial adaptation among left-behind children. Therefore, Hypothesis 1 (H1) was proposed: External assets are positively associated with psychosocial adaptation among left-behind children.

### 1.2 Mediating role of stress mindset

Stress mindset refers to an individual’s overarching belief regarding whether stress has enhancing or debilitating effects. It comprises two dimensions: stress-is-enhancing and stress-is-debilitating mindsets. Individuals who endorse the stress-is-enhancing mindset believe that stress can improve cognitive performance, psychological functioning, and related outcomes, whereas those who endorse the stress-is-debilitating mindset believe that it undermines these outcomes (Crum et al., 2013). According to the social cognitive processing model (Lepore and Revenson, 2007), supportive environments influence developmental outcomes by shaping individuals’ subjective interpretations of adverse circumstances. When facing adversity, individuals who receive social support are more likely to interpret unfavorable circumstances positively, fostering a stress-is-enhancing mindset. Social support, as a component of external assets, facilitates individuals’ positive interpretation of adverse circumstances and fosters a stress-is-enhancing mindset (Wu et al., 2024). Furthermore, individuals endorsing a stress-is-enhancing mindset are more likely to appraise stress as a challenge rather than a threat when encountering adverse circumstances, adopt more proactive coping strategies, experience fewer negative emotions such as anxiety and depression, and ultimately achieve better developmental outcomes (Bosshard and Gomez, 2024; Schreiber and Schotanus-Dijkstra, 2024). At the physiological level, evidence indicates that an individual’s stress mindset influences cortisol responses under acute stress (Klatzkin et al., 2025). Individuals with a stress-is-enhancing mindset exhibit more adaptive physiological responses to stress, which may contribute to positive psychological and behavioral adaptation by regulating the hypothalamic–pituitary–adrenal (HPA) axis (Langrafe Junior et al., 2025). It can therefore be inferred that, when left-behind children gain more external resources from family, school, and community, they are more likely to interpret the situation positively, viewing left-behind life as an opportunity for growth, adopting a stress-is-enhancing mindset, and exhibiting better psychosocial adaptation. Accordingly, Hypothesis 2 (H2) was proposed: Stress mindset mediates the association between external assets and psychosocial adaptation among left-behind children.

### 1.3 Moderating role of environmental sensitivity

Environmental sensitivity may moderate the indirect effects of external assets on left-behind children’s psychosocial adaptation. According to relational developmental systems theory, individual development results from interactions between the individual and the external environment. However, the extent to which individuals are influenced by the external environment varies with their environmental sensitivity (Assary et al., 2024). Environmental sensitivity refers to individuals’ capacity to register and process environmental stimuli (Pluess et al., 2018). The differential susceptibility model posits that individuals with high environmental sensitivity perceive and process environmental stimuli more deeply; they are more susceptible to the adverse effects of negative environments and more likely to benefit from positive environments (Belsky and Pluess, 2009; Lionetti and Pluess, 2024), which is primarily manifested in emotional states and physiological responses (Aron et al., 2012). Previous studies have shown that, among children with higher environmental sensitivity, the positive association between family support (a component of external assets) and physical and mental health is stronger (Scrimin et al., 2018); school-based anti-bullying programs (a component of external assets) are associated with greater improvements in bullying prevention skills and social competence (Acevedo et al., 2025); and improvements in neighborhood safety (a component of external assets) are more strongly associated with social adaptation (Cai et al., 2024). Based on the Differential Susceptibility Model, greater external assets may be more strongly associated with positive developmental outcomes among left-behind children with higher environmental sensitivity. Accordingly, Hypothesis 3 was proposed: Environmental sensitivity moderates the association between external assets and psychosocial adaptation; specifically, this association is stronger among left-behind children with higher levels of environmental sensitivity.

Environmental sensitivity amplifies an individual’s response to external environmental stimuli and enhances the perception and processing of the internal psychological environment (Lionetti and Pluess, 2024). Compared to low-sensitivity individuals, highly sensitive individuals exhibit fewer emotional and behavioral problems and higher levels of mental health when they adopt positive emotion regulation strategies, such as cognitive reappraisal and refocusing on planning (Džida et al., 2024; Yano and Oishi, 2024). Stress mindset is conceptually similar to positive emotion regulation strategies in that both involve the positive construction of meaning in response to adversity. Therefore, when highly environmentally sensitive left-behind children endorse a stress-is-enhancing mindset, their positive meaning-making of adverse circumstances is processed more deeply, ultimately leading to better psychosocial adaptation. Accordingly, Hypothesis 4 was proposed: Environmental sensitivity moderates the association between stress mindset and psychosocial adaptation; specifically, this association is stronger among left-behind children with higher levels of environmental sensitivity.

**FIGURE 1:**
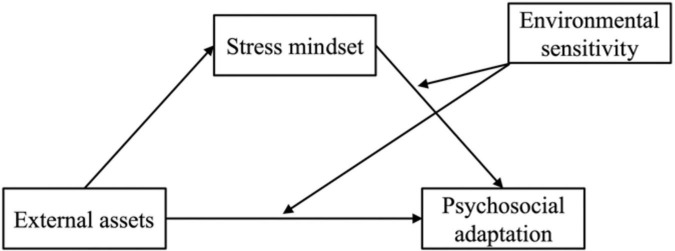
Moderated mediation hypothesis model.

Additionally, left-behind primary school students exhibited higher levels of psychosocial adaptation than their junior high school counterparts, and male students exhibited higher levels than female students (Fan et al., 2024). Accordingly, grade and sex were included as covariates in this study. Drawing on the Positive Youth Development perspective, this study establishes a moderated mediation model (Figure 1) to explore the association between external assets and psychosocial adaptation in left-behind children. Specifically, it examines the mediating effect of stress mindset and the moderating influence of environmental sensitivity, thereby clarifying how external assets are linked to psychosocial adaptation and the conditions under which this association may vary. These findings may offer empirical evidence and theoretical insights to inform the development of effective intervention strategies for this demographic.

## 2 Materials and methods

### 2.1 Participants and procedure

Stratified cluster random sampling was employed for participant recruitment between March and July 2025. Initially, five counties in the Yunnan Province were selected to capture diverse rural contexts and ensure broad geographical coverage. Subsequently, a lottery method was used to randomly select one primary school and one junior high school from the townships within each county, yielding 10 schools (five primary and five junior high schools). As this study assessed abstract psychological constructs, and previous evidence suggests that children under 10 years of age exhibit relatively limited comprehension and response consistency on self-report Likert scales (Mellor and Moore, 2014), the target population was restricted to students in grades 4–9 (aged 10–15 years). Within these schools, two classes per eligible grade were randomly selected via lottery, and all students in the selected classes were invited to participate.

Before data collection, all investigators underwent standardized training to ensure procedural consistency. Questionnaires were administered collectively on a class-by-class basis. Through an on-site distribution and immediate collection procedure, the investigators provided standardized verbal instructions, explicitly emphasizing the principles of independent response, voluntary participation, and data confidentiality.

The inclusion criteria for left-behind children were as follows: (1) aged 10–15 years; and (2) one or both parents had migrated for work for a duration of 6 months or longer. Initially, 2,865 questionnaires were distributed. After excluding invalid surveys—specifically those with missing data on key variables, uniform answers, or obvious patterned responding—2,639 valid questionnaires were retained, yielding an effective response rate of 92.11%. The final sample comprised 1,590 left-behind children and 1,049 non-left-behind children. The demographic distribution of the participants according to sex and grade is presented in Table 1.

**TABLE 1:** Participant profile.

Characteristics	Total (*N* = 2,639)	Left-behind children (*n* = 1,590)	Non-left-behind children (*n* = 1,049)
Sex			
Male	1,202 (45.5%)	709 (44.6%)	493 (47.0%)
Female	1,437 (54.5%)	881 (55.4%)	556 (53.0%)
Grade			
Primary school	1,187 (45.0%)	702 (44.2%)	485 (46.2%)
Junior high school	1,452 (55.0%)	888 (55.8%)	564 (53.8%)

### 2.2 Measurements

#### 2.2.1 External assets

External assets were assessed using the external assets subscale of the Developmental Assets Profile (DAP), originally developed by Search Institute (2005) and subsequently revised by Syvertsen et al. (2021). In accordance with the recent revision, the extracurricular activity participation dimension was excluded from the factor structure because it measures behavioral frequency rather than a latent construct and exhibits suboptimal psychometric properties. The revised subscale employed in this study comprised 29 items and three factors: support, mattering, belonging, and boundaries. For example, one item states: “In my school, there are clear rules about what students can and cannot do.” Each item is rated on a 5-point Likert scale, except for item 53, which uses a 6-point scale. Higher total scores reflect greater external assets. In the present study, the Cronbach’s alpha coefficient for the subscale was 0.810.

#### 2.2.2 Stress mindset

Stress mindset was measured using the Stress Mindset Measure (SMM) developed by Crum et al. (2013) and revised by Wang et al. (2023). The scale included eight items and two factors: stress-is-enhancing mindset and stress-is-debilitating mindset. An example item is:“Experiencing stress enhances my performance and productivity.” Each item is rated on a 5-point Likert scale ranging from 0 to 4. After reverse-scoring the stress-is-debilitating items, higher total scores indicated a stronger stress-is-enhancing mindset. In this study, Cronbach’s alpha coefficient for the scale was 0.928.

#### 2.2.3 Environmental sensitivity

Environmental sensitivity was measured using the Highly Sensitive Child Scale–21 item version (HSC-21) revised by Weyn et al. (2022). The scale contains 21 items and includes two specific factors: Ease of Excitation–Low Sensory Threshold (EOE-LST) and Aesthetic Sensitivity (AES). For example: “I notice when small things have changed in my environment.” EOE-LST reflects the extent to which individuals are easily activated or overwhelmed by intense, complex, or excessive stimuli, while AES reflects individuals’ awareness of subtle, meaningful, or aesthetically relevant environmental cues. These two dimensions represent specific expressions of the same sensitivity trait under different environmental cues, and, taken together, they converge on an overarching tendency for individuals to perceive and process environmental information at a deeper level (Weyn et al., 2022; Lionetti and Pluess, 2024). Previous psychometric evidence supports using the HSC-21 total score to indicate overall environmental sensitivity (Weyn et al., 2022). Items were rated on a 7-point Likert scale, with higher scores indicating higher sensitivity. Cronbach’s α coefficient for the scale was 0.863.

#### 2.2.4 Psychosocial adaptation

Psychosocial adaptation is conceptualized as a multidimensional construct and a key indicator of mental health; nonetheless, no standardized instrument has been widely adopted to assess it. Previous research in this demographic has primarily focused on maladaptive outcomes among left-behind children, such as depression and externalizing problem behaviors, with less attention given to positive adaptation (Fan and Wu, 2020; Yang and Huang, 2022). The dual-factor model of mental health posits that adaptation represents a holistic state encompassing both negative and positive dimensions. The positive youth development perspective further emphasizes that the mere absence of maladjustment does not necessarily indicate a good level of adaptation. Recent empirical evidence suggests that, alongside negative indicators like depression, externalizing problems, and peer rejection, left-behind children also exhibit positive adjustment, including happiness, prosocial behavior, and peer acceptance (Liu et al., 2023, 2024). Therefore, drawing on established assessment approaches (Hou et al., 2022; Fan et al., 2023), this study selected these six specific indicators to provide a comprehensive, multidimensional evaluation of psychosocial adaptation among left-behind children.

##### 2.2.4.1 Happiness

Happiness was measured using the 8-item short-form of the Oxford Happiness Questionnaire (OHQ-SF) developed by Hills and Argyle (2002), and later revised by Li and Chen (2013). An example item is: “I feel that life is very rewarding.” Each item is rated on a 6-point Likert scale ranging from 1 to 6. Higher total scores indicated higher levels of happiness. In this study, Cronbach’s alpha coefficient for the scale was 0.680.

##### 2.2.4.2 Depression

Depression was measured using the 10-item short form of the Center for Epidemiological Studies Depression Scale (CES-D-10), developed by Andresen et al. (1993), and revised by Yang et al. (2018). The scale included 10 items and three factors: depressed affect, positive affect, and somatic symptoms. For example: “I had trouble keeping my mind on what I was doing.” Each item is rated on a 4-point Likert scale (0–3) to assess the frequency of symptoms experienced during the past week. After reverse-scoring two items, higher total scores indicated more severe levels of depression. In this study, Cronbach’s alpha coefficient for the scale was 0.724.

##### 2.2.4.3 Prosocial behavior

Prosocial behavior was measured using the Prosocial Tendencies Measure (PTM) developed by Carlo and Randall (2002) and later revised by Kou et al. (2007). The scale included 26 items and six factors: public, anonymous, altruistic, compliant, emotional, and dire prosocial tendencies. An example item is: “When others ask me for help, I rarely refuse.” Each item is rated on a 5-point Likert scale ranging from 1 to 5. Higher total scores indicated stronger prosocial tendencies. In this study, Cronbach’s alpha coefficient for the scale was 0.919.

##### 2.2.4.4 Externalizing problems

Externalizing problems were measured using the Externalizing Problems subscale of the Youth Self-Report (YSR), which is the adolescent self-report version of the Child Behavior Checklist (CBCL) developed by Achenbach and Rescorla (2001) and revised by Wang et al. (2013). The subscale included 32 items and two factors: rule-breaking behavior and aggressive behavior. For example: “I truant, skip school.” Each item is rated on a 3-point Likert scale ranging from 0 to 2. Higher total scores indicated more severe externalizing problems. In this study, Cronbach’s alpha coefficient for the subscale was 0.819.

##### 2.2.4.5 Peer acceptance

Peer acceptance was measured using the Peer Acceptance subscale of the Peer Relationship Scale developed by Zou et al. (1998). The subscale included 20 items. An example item is: “It is relatively easy for me to make friends at school.” Each item is rated on a 4-point Likert scale ranging from 1 to 4. Higher total scores indicated better peer relationships. In this study, Cronbach’s alpha coefficient for the subscale was 0.868.

##### 2.2.4.6 Peer rejection

Peer rejection was measured using the Fear and Inferiority subscale of the Peer Relationship Scale developed by Zou et al. (1998). The subscale included 10 items. An example item is: “I feel that some classmates often make fun of me.” Each item is rated on a 4-point Likert scale ranging from 1 to 4. Higher total scores indicated higher levels of peer rejection. In this study, Cronbach’s alpha coefficient for the subscale was 0.819.

In summary, the six indicators—depression, happiness, externalizing problem behaviors, prosocial behavior, peer rejection, and peer acceptance—jointly represent the latent construct of psychosocial adaptation among left-behind children. Following the psychometric recommendations for composite outcome measures (Moreau and Wiebels, 2021), theoretically related indicators were combined into a weighted composite score. This approach integrates shared information across individual measures, reduces measurement-specific error, and thus provides a more precise estimate of the latent construct. Compared with more complex latent variable models, the composite score also offers higher transparency and interpretability. Furthermore, because the six indicators may contribute differently to psychosocial adaptation, this study followed Fan et al. (2024) by assigning weights based on each indicator’s factor loading on the first common factor. This ensures that indicators contributing more to the latent psychosocial adaptation construct receive higher weights in the composite score. The resulting weighted composite score was subsequently used to test the hypothesized mediation and moderated mediation models, thereby ensuring that the weight determination is independent of the model outcomes.

To evaluate whether these six indicators were suitable for being combined into a single overall index, Bartlett’s test of sphericity and the Kaiser–Meyer–Olkin (KMO) measure were first examined. Bartlett’s test of sphericity yielded a value of 4702.62 (*p* < 0.001), and the KMO measure was 0.814, indicating that these indicators were appropriate for composite construction. Exploratory factor analysis using principal component analysis was then conducted on the six indicators. The results showed that only one factor with an eigenvalue greater than 1 was extracted (eigenvalue = 3.08), accounting for 51.35% of the total variance. Based on the factor loadings, Z-scores of the variables, and the eigenvalue, the composite score for psychosocial adaptation was calculated as follows:


Psychosocial⁢adaptation=

$$\frac{\begin{array}{c} 0.785\,Z_{\text{happiness}}+0.675\,Z_{\mathrm{prosocial}\,\mathrm{behavior}}+0.777\,Z_{\mathrm{peer}\,\mathrm{acceptance}}\\ -0.716\,Z_{\text{depression}}-0.627\,Z_{\mathrm{externalizing}\,\mathrm{problems}}-0.707\,Z_{\mathrm{peer}\,\mathrm{rejection}} \end{array}}{3.08}$$

The resulting scores ranged from −4.24 to 2.88, with higher scores indicating better psychosocial adaptation.

### 2.3 Statistical analysis

All statistical analyses were performed using SPSS version 27.0. First, common method bias was examined using both Harman’s single-factor test and the common latent factor (CLF) approach. Second, descriptive statistics and Pearson’s correlation analyses were conducted for the main variables. Third, independent-samples t-tests were used to examine differences between left-behind and non-left-behind children, whereas two-way ANOVAs were used to assess the effects of sex and grade among left-behind children. Fourth, the PROCESS macro (Version 4.0) was used to test the hypothesized models, with Model 4 used for the mediation model and Model 15 for the moderated mediation model. A bias-corrected percentile bootstrap method with 5,000 resamples was used to test the significance of the effects; 95% confidence intervals (CIs) that excluded 0 indicated statistical significance. Significant interactions were further probed using simple slope analysis.

Two sets of supplementary analyses were conducted. First, given the slightly low internal consistency of the happiness measure (α = 0.680) and the limited reliability of the depression measure (α = 0.724), sensitivity analyses were conducted on these two indicators to ensure that the conclusions drawn from the composite psychosocial adaptation score were robust. Second, given that the two dimensions of environmental sensitivity may capture different aspects of sensitivity, dimension-level moderated mediation analyses were conducted, using ease of excitation–low sensory threshold and aesthetic sensitivity as moderators, separately.

## 3 Results

### 3.1 Common method bias test

To mitigate potential common method bias (CMB), procedural controls were implemented during data collection, including anonymous administration and reverse-scoring of certain items. Two methods were used to examine potential CMB.

First, Harman’s single-factor test was conducted using exploratory factor analysis. The results extracted 43 factors with eigenvalues greater than one, and the first unrotated factor explained only 11.4% of the total variance, which was below the commonly used threshold of 40% (Podsakoff and Organ, 1986), suggesting that the common method bias problem was not serious.

Second, according to the common latent factor (CLF) approach (Podsakoff et al., 2003; Podsakoff et al., 2024), we constructed a constrained CLF model by adding a constrained common method factor to the benchmark measurement model. The constrained CLF model fit the data well (CFI = 0.982, TLI = 0.969, RMSEA = 0.058, SRMR = 0.036). Compared with the benchmark model (CFI = 0.978, TLI = 0.964, RMSEA = 0.062, SRMR = 0.040), the addition of the common method factor only slightly improved model fit (ΔCFI = 0.003, ΔTLI = 0.004, ΔRMSEA = −0.004, ΔSRMR = −0.004). The changes in CFI and TLI were both below the recommended threshold of 0.02, indicating that the common method factor did not substantially improve the benchmark model.

To further evaluate the potential impact of CMB, we used the unmeasured latent method factor (ULMC) model. The results showed that the normalized load squares for method factor pairs were all below the 20% criterion, with an average of 13.7%. The absolute changes in the standardized critical path coefficients were all below the 10% criterion, ranging from 2.7 to 9.4%. Overall, these results indicate that CMB did not substantially affect the conclusions. Future studies could further mitigate potential bias using multi-wave, multi-informant, or multi-source data collection.

### 3.2 Differences in study variables between left-behind and non-left-behind children

Descriptive statistics for the study variables among left-behind and non-left-behind children are presented in Table 2. Independent-samples t-tests revealed that left-behind children scored significantly lower on external assets, stress mindset, and psychosocial adaptation than their non-left-behind peers. However, no significant group differences were observed for environmental sensitivity. Consequently, non-left-behind children, who served as the control group, were excluded from subsequent analyses.

**TABLE 2:** Scores of study variables for left-behind and non-left-behind children (x̄±s).

Variables	Total (*N* = 2,639)	Left-behind children (*n* = 1,590)	Non-left-behind children (*n* = 1,049)	*t* (*df* = 2,637)
External assets	3.609 ± 0.411	3.517 ± 0.409	3.750 ± 0.373	−14.847***
Stress mindset	2.111 ± 0.805	2.078 ± 0.826	2.160 ± 0.772	−2.566**
Environmental sensitivity	4.560 ± 0.774	4.573 ± 0.778	4.540 ± 0.767	1.073
Psychosocial adaptation	0.000 ± 1.000	−0.122 ± 1.010	0.184 ± 0.956	−7.870***

**p < 0.01,

***p < 0.001, the same below.

### 3.3 Correlation analysis of research variables in left-behind children

A two-way analysis of variance (ANOVA) was conducted to examine whether there were differences in the psychosocial adaptation of left-behind children across sexes and grades. The results indicated that the main effects of sex [F_(1,1586)_ = 6.36, *p* < 0.05, $\eta_{\text{p}}^{2}=0.004$] and grade [F_(1,1586)_ = 35.04, *p* < 0.001,$\eta_{\text{p}}^{2}=0.022$] were significant, whereas the interaction effect between sex and grade was not significant. Further analysis revealed that the psychosocial adaptation scores of male left-behind children (-0.032 ± 0.997) were significantly higher than those of females (-0.193 ± 1.015). Similarly, primary school students exhibited significantly higher psychosocial adaptation scores (0.055 ± 1.031) compared to junior high school students (-0.261 ± 0.972).

As shown in Table 3, Pearson correlation analysis demonstrated significant pairwise correlations between external assets, stress mindsets, environmental sensitivity, and psychosocial adaptation in left-behind children. Furthermore, sex and grade were significantly correlated with psychosocial adaptation. Consequently, sex and grade were included as control variables in the subsequent regression analyses.

**TABLE 3:** Descriptive statistics and correlation analysis of research variables.

Variables	1	2	3	4	5	6
1. External assets	1	1	1	1	1	1
2. Stress mindset	0.28***					
3. Environmental sensitivity	0.05*	−0.09***				
4. Psychosocial adaptation	0.38***	0.48***	−0.19***			
5. Sex	−0.05	−0.01	0.09***	−0.08**		
6. Grade	−0.14***	−0.05*	0.01	−0.16***	0.10***	

*N* = 1,590. Sex is a dummy variable, male = 0, female = 1. Grade is a dummy variable, primary school students = 0, junior high school students = 1.

*p < 0.05,

**p < 0.01,

***p < 0.001.

### 3.4 Moderated mediation effect analysis

Initially, using Model 4 from the SPSS macro program PROCESS, which represents a simple mediation model, the mediating role of stress mindset on the relationship between external assets and psychosocial adaptation was examined while controlling for sex and grade.

As shown in Table 4, external assets were significantly and positively associated with psychosocial adaptation (β = 0.360, *t* = 15.439, *p* < 0.001) and stress mindset (β = 0.278, *t* = 11.438, *p* < 0.001). Thus, Hypothesis 1 is supported. When external assets and stress mindset were simultaneously entered into the regression model with psychosocial adaptation as the outcome variable, stress mindset showed a significant positive association with psychosocial adaptation (β = 0.404, *t* = 18.516, *p* < 0.001). Moreover, the direct association between external assets and psychosocial adaptation remained significant (β = 0.247, *t* = 11.249, *p* < 0.001). The bias-corrected percentile bootstrap test (with 5,000 resamples) indicated that the indirect effect of stress mindset was 0.112 (Boot *SE* = 0.012), and its 95% CI was [0.090, 0.135], excluding 0, indicating a significant mediating effect of stress mindset. The proportion of this mediation effect was 31%. This finding suggests that stress mindset partially mediates the relationship between external assets and psychosocial adaptation, supporting Hypothesis 2.

**TABLE 4:** Mediation model test of stress mindset.

Predictor variable	Model 1 (psychosocial adaptation)			Model 2 (stress mindset)			Model 3 (psychosocial adaptation)		
	β	*SE*	95%CI	β	*SE*	95%CI	β	*SE*	95%CI
Sex	−0.105*	0.047	[−0.196, −0.013]	0.015	0.049	[−0.081, 0.110]	−0.111**	0.042	[−0.194, −0.028]
Grade	−0.203***	0.047	[−0.296, −0.111]	−0.033	0.049	[−0.129, 0.064]	−0.190***	0.043	[−0.274, −0.106]
External assets	0.360***	0.023	[0.314, 0.406]	0.278***	0.024	[0.231, 0.326]	0.247***	0.022	[0.204, 0.291]
Stress mindset	0.155			0.079			0.404***	0.022	[0.361, 0.447]
*R* ^2^							0.305		
*F*	97.019***			45.234***			174.154***		

*p < 0.05,

**p < 0.01,

***p < 0.001.

**FIGURE 2:**
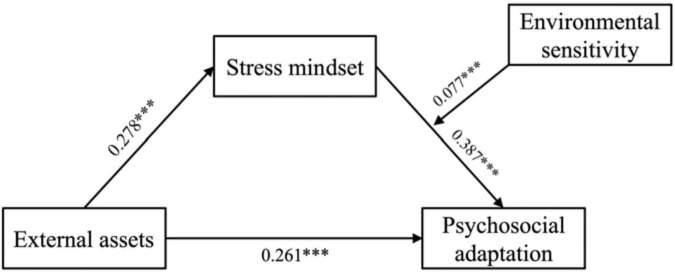
Moderated mediation model of stress mindset and environmental sensitivity in the association between external assets and psychosocial adaptation. ***p < 0.001.

Subsequently, Model 15 of the SPSS PROCESS macro (which assumes that the moderating variable influences the second half of the mediation process and the direct path, which is consistent with the theoretical hypotheses of this study) was used to examine the moderating effect of environmental sensitivity. By controlling for sex and grade, the analysis aimed to determine whether the effect of stress mindset was moderated by environmental sensitivity. As shown in Table 5, the interaction term between external assets and environmental sensitivity was not significantly associated with psychosocial adaptation (β = −0.007, *t* = −0.362, *p* > 0.05). Thus, environmental sensitivity did not moderate the direct path from external assets to psychosocial adaptation, refuting Hypothesis 3. However, the interaction term between stress mindset and environmental sensitivity was significantly and positively associated with psychosocial adaptation (β = 0.077, *t* = 4.101, *p* < 0.001). This indicates that environmental sensitivity significantly moderated the relationship between stress mindset and psychosocial adaptation, specifically in the second half of the mediation model, supporting Hypothesis 4. The complete moderated mediation model, including all standardized path coefficients, is shown in Figure 2.

**TABLE 5:** Testing for moderated mediation model.

Predictor variable	Model 1 (stress mindset)			Model 2 (psychosocial adaptation)		
	β	*SE*	95%CI	β	*SE*	95%CI
Sex	0.015	0.049	[−0.081, 0.110]	−0.074	0.042	[−0.156, 0.008]
Grade	−0.033	0.049	[−0.129, 0.064]	−0.195***	0.042	[−0.278, −0.113]
External assets	0.278***	0.024	[0.231, 0.326]	0.261***	0.022	[0.219, 0.303]
Stress mindset	0.079			0.387***	0.022	[0.345, 0.429]
Environmental sensitivity				−0.162***	0.021	[−0.202, −0.121]
External assets × environmental sensitivity				−0.007	0.020	[−0.047, 0.032]
Stress mindset × environmental sensitivity				0.077***	0.019	[0.040, 0.113]
*R* ^2^				0.338		
*F*	45.234***			115.193***		

***p < 0.001.

The indirect effect of external assets on psychosocial adaptation through stress mindset was moderated by environmental sensitivity. The index of moderated mediation was 0.108 (95% CI [0.086, 0.130]), supporting the moderated mediation model. Conditional indirect effects at different levels of environmental sensitivity are presented in Table 6. The indirect effect was stronger for left-behind children with high environmental sensitivity (β = 0.129, *SE* = 0.014, 95% CI [0.103, 0.157]) than for those with low environmental sensitivity (β = 0.086, *SE* = 0.010, 95% CI [0.067, 0.107]).

**TABLE 6:** Mediating effect values of stress mindset across different environmental sensitivity levels.

Mediating variable	Environmental sensitivity	β	*SE*	95% CI
Stress mindset	*M*-*SD*	0.086	0.010	[0.067, 0.107]
	*M*	0.108	0.011	[0.086, 0.130]
	*M+SD*	0.129	0.014	[0.103, 0.157]

**FIGURE 3:**
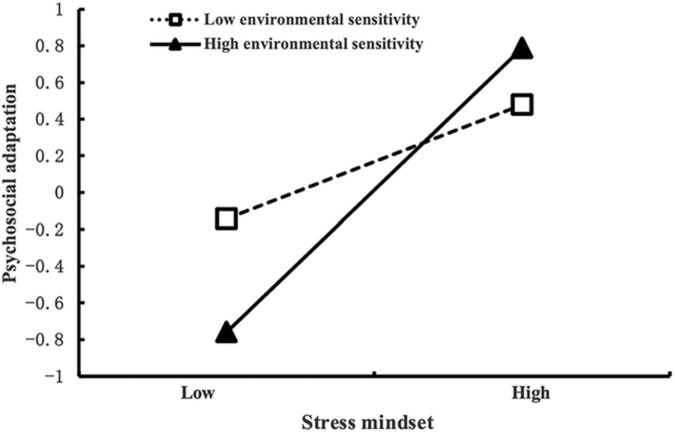
The moderating role of environmental sensitivity between stress mindset and psychosocial adaptation.

To further elucidate the nature of this interaction, a simple slope analysis was conducted at high (*M* + 1 *SD*) and low (*M* − 1 *SD*) levels of environmental sensitivity, as depicted in Figure 3. The results showed that when environmental sensitivity was low, the positive association between stress mindset and psychosocial adaptation was significant (*B*_*simple*_ = 0.310, *t* = 10.994, *p* < 0.001). When environmental sensitivity was high, this positive association remained significant and was stronger (*B*_*simple*_ = 0.464, *t* = 16.187, *p* < 0.001). Overall, these findings suggest that the positive association between stress mindset and psychosocial adaptation was stronger among left-behind children with higher levels of environmental sensitivity.

### 3.5 Sensitivity analyses

To examine whether the findings based on the composite psychosocial adaptation score were robust when individual indicators were considered separately, sensitivity analyses were conducted focusing on depression and happiness, which represent the negative and positive dimensions of adaptation, and showed relatively low internal consistency.

As indicated in Supplementary Table 1, the results closely aligned with those obtained from the composite score. When depression was analyzed as the outcome, the interaction between external assets and environmental sensitivity was not significantly associated with depression (β = −0.003, *p* > 0.05). In contrast, the interaction between stress mindset and environmental sensitivity demonstrated a significant negative association with depression (β = −0.100, *p* < 0.001). The bias-corrected percentile bootstrap test showed that the index of moderated mediation was −0.028 (Boot *SE* = 0.006), with a 95% confidence interval of [−0.041, −0.016] that did not include zero. Similarly, when happiness was analyzed as the outcome, the interaction between external assets and environmental sensitivity was not significantly associated with happiness (β = 0.007, *p* > 0.05), whereas the interaction between stress mindset and environmental sensitivity was significantly and positively associated with happiness (β = 0.052, *p* < 0.01). The bias-corrected percentile bootstrap test indicated that the index of moderated mediation was 0.014 (Boot *SE* = 0.006), with a 95% confidence interval of [0.004, 0.027], excluding 0. Collectively, these findings indicate that the primary conclusions based on the composite psychosocial adaptation score remained robust when these representative positive and negative indicators were analyzed separately.

Given the bifactor nature of the HSC-21, supplementary factor-level analyses were further conducted using ease of excitation–low sensory threshold and aesthetic sensitivity as separate moderators. As shown in Supplementary Table 2, the results were consistent with those based on the HSC-21 total score. Specifically, the interactions between external assets and each HSC-21 factor were not significant, whereas the interactions between stress mindset and each HSC-21 factor were significant. The indices of moderated mediation were also significant for both factors. These findings suggest that the moderated mediation pattern was not driven solely by one specific dimension of environmental sensitivity but was generally consistent across both specific factors.

## 4 Discussion

Grounded in the Positive Youth Development perspective, this study reveals the complex mechanisms linking external assets to psychosocial adaptation among left-behind children. Specifically, it highlights the mediating effect of stress mindset and the moderating influence of environmental sensitivity. These findings underscore how ecological contexts and individual psychological traits synergistically shape adaptation, offering a nuanced theoretical framework for understanding this population.

### 4.1 Differences in study variables between left-behind and non-left-behind children

The results of this study indicated that levels of external assets and psychosocial adaptation among left-behind children were significantly lower than among non-left-behind children, suggesting that the experience of being left behind may be linked to developmental disadvantages. According to family functioning theory, sound family functioning is an important contextual factor for children’s physical and mental health development (Fang et al., 2004). However, in the context of parental absence, insufficient parental supervision and guidance, combined with impaired parent-child relationships, may be associated with a higher risk of emotional distress and externalizing problems (Shao et al., 2018), which may help explain the lower levels of psychosocial adaptation observed among left-behind children. Notably, although left-behind children possess fewer external assets than non-left-behind children, they are not in a state of “resource depletion.” Therefore, the deficit perspective associated with left-behind status should not be overstated, nor should this population be viewed solely through a problem-focused lens. Instead, attention should be directed toward the developmental assets and growth potential that these children maintain despite adverse family circumstances.

### 4.2 Association between external assets and the psychosocial adaptation of left-behind children

After controlling for the effects of grade and sex, external assets were significantly associated with psychosocial adaptation among left-behind children, supporting Hypothesis 1. This finding is consistent with prior research on developmental assets, which has shown that a greater number of assets is associated with more positive developmental outcomes (Chang and Zhang, 2013). Furthermore, this study extends the applicability of developmental asset theory to left-behind children, suggesting that even in adverse family circumstances, external assets may be linked to better psychosocial adaptation. Although left-behind children experience a relative lack of parental companionship, when they can access multiple external developmental resources—such as remote parental involvement, grandparental care, school support, and neighborhood support—the integration of resources across different ecological domains may help buffer risks associated with adverse family contexts and may be associated with higher levels of psychosocial adaptation.

### 4.3 Mediating role of stress mindset

Regarding the mediating pathway, stress mindset partially mediated the association between external assets and psychosocial adaptation among left-behind children, supporting Hypothesis 2. This result aligns with the social cognitive processing model (Lepore and Revenson, 2007), suggesting that external assets may be associated with individuals’ adaptation by shaping how they appraise stressful experiences. For left-behind children, parental migration may constitute a chronic stressor, involving parent–child separation, reduced emotional companionship, and daily-life challenges. In this context, greater external assets from family, school, and community contexts may provide not only practical support but also a constructive interpretive framework. This framework may help children view parental migration and related difficulties as manageable challenges rather than uncontrollable threats, which may be associated with a higher level of stress-is-enhancing mindset. This finding is consistent with Wu et al. (2024), who reported that social support was related to a stress-is-enhancing mindset among healthcare workers.

Regarding the latter part of the mediating pathway, the present study found that a stress-is-enhancing mindset was associated with better psychosocial adaptation among left-behind children. This finding is consistent with prior research showing that individuals who endorse a stress-is-enhancing mindset tend to report lower perceived stress, more positive emotions, more adaptive coping, and better psychological and academic outcomes (Keech et al., 2018; Williams and Ginty, 2024). In the left-behind context, this mindset may help children view left-behind experiences as opportunities for growth, be more inclined to cope with challenges independently, and adopt positive coping strategies, thereby showing better psychosocial adaptation. Overall, stress mindset may be a key mechanism linking external developmental assets to adaptive outcomes in left-behind children.

### 4.4 Moderating role of environmental sensitivity

The results of this study indicate that environmental sensitivity did not moderate the association between external assets and psychosocial adaptation; thus, Hypothesis 3 was not supported. This unexpected finding may reflect the coexistence of external resources and developmental risks in the context of parental migration. External assets provided by the family, school, and community may serve as positive environmental cues, whereas prolonged parental absence may constitute salient negative cues. From the perspective of the Differential Susceptibility Model, highly sensitive children may respond to both types of cues rather than only to supportive resources (Belsky and Pluess, 2009; Pluess and Belsky, 2013; Lionetti and Pluess, 2024). Thus, for highly sensitive left-behind children, the benefits of resource-related cues may be offset by vulnerabilities activated by risk-related cues, which may explain why environmental sensitivity did not significantly moderate the association between external assets and psychosocial adaptation. However, this interpretation remains a *post-hoc* theoretical speculation and should be prospectively tested in future longitudinal or experimental research.

Environmental sensitivity significantly moderated the association between stress mindset and psychosocial adaptation, supporting Hypothesis 4. Specifically, compared to left-behind children with low environmental sensitivity, the positive association between a stress-is-enhancing mindset and psychosocial adaptation was stronger among those with high environmental sensitivity. This finding is consistent with Environmental Sensitivity Theory and prior empirical evidence—highly sensitive individuals tend to engage in deeper perception and processing of internal psychological cues and may be more responsive to them (Lionetti and Pluess, 2024; Yano and Oishi, 2024). In the context of being left behind, highly sensitive children who endorse a stress-is-enhancing mindset may be more inclined to interpret parental migration, an adverse circumstance, as an opportunity for personal growth, which may be associated with a more positive internal psychological environment. Furthermore, as highly sensitive children process cognitive cues more deeply, constructive meaning-making may be more salient and more strongly linked to positive cognitive patterns. This process may be associated with the adoption of proactive coping strategies and better psychosocial adaptation. Taken together, these findings may suggest that the amplifying role of environmental sensitivity is not uniform. From a *post-hoc* theoretical perspective, such amplification may be interpreted as more likely to emerge when the processed cues are internally consistent and positively oriented, but less evident when children are exposed to mixed or competing environmental cues. This possibility should be examined in future research.

Reflecting the bifactor model of the HSC-21, although the total score represents overall environmental sensitivity, the scale intrinsically captures two specific factors: Ease of Excitation–Low Sensory Threshold and Aesthetic Sensitivity. Consistently, the supplementary factor-level analyses revealed patterns aligned with those from the total score, suggesting that the main moderated mediation findings were not driven solely by a single factor. Nevertheless, future research should further examine whether these two factors operate differently across environmental contexts and developmental outcomes.

### 4.5 Implications

This study revealed the relationship between external assets and psychosocial adaptation, as well as the mediating role of stress mindset and the moderating role of environmental sensitivity, through a survey of 1,590 rural left-behind children. By shifting the focus from the traditional deficit model to a strength-building perspective, this study significantly expands the application of the PYD framework within this vulnerable demographic. Consequently, these findings provide preliminary empirical evidence that may inform the development of targeted multitiered intervention strategies. The following actionable recommendations are proposed for policymakers, schools, and families to support psychosocial adaptation among these children.

First, at the policy level, special funding should be considered to support community-based programs that promote psychosocial adaptation. Specifically, community-level interventions—such as coping skills training, emotion regulation programs, and peer support groups—could be developed and evaluated to support left-behind children in developing a stress-is-enhancing mindset.

Second, in the educational context, schools could consider specialized teacher training to help educators identify students with lower levels of psychosocial adaptation. Educators might also incorporate cognitive reframing activities to help left-behind children reflect on possible growth-related aspects of their experiences, which may support a stress-is-enhancing mindset. Additionally, future school-based programs could consider highly sensitive children by providing more individualized emotional support and evaluating whether such support benefits this subgroup.

Finally, regarding the family environment, parents may need to recognize that children’s interpretations of the left-behind experience may be related to their adaptation. Therefore, parents might consider communicating about migration in ways that may help children view the experience as manageable rather than purely negative. Caregivers may also need to be attentive to emotional conflict in the family environment, particularly for children with higher environmental sensitivity. Collectively, these implications may help inform the development of supportive environments for left-behind children, but their effectiveness should be tested through longitudinal and intervention research.

### 4.6 Limitation

This study has several limitations that warrant consideration. First, the cross-sectional design precludes causal inferences regarding the relationships among variables. Future studies should adopt longitudinal designs to clarify the temporal order and causal directionality of these associations.

Second, the participants were recruited exclusively from rural schools in Yunnan Province, which may limit the external validity of the findings. Yunnan Province has unique geographic characteristics, urban–rural socioeconomic disparities, and a relatively high proportion of ethnic minority populations. These contextual characteristics may influence children’s opportunities to obtain external assets, their interpretations of parental migration for work, and their levels of psychosocial adaptation. Previous empirical studies among left-behind children in Yunnan and southwest ethnic regions also suggest that local social, familial, and ethnic contexts may shape psychosocial adaptation patterns (Tian et al., 2019; Ran et al., 2024; Zhuang et al., 2024). In particular, stress mindset may be shaped by ethnic cultural values and family norms, which may vary across ethnic groups. Because this study did not include ethnic background as a core variable or further compare developmental patterns between Han children and ethnic minority children, caution should be exercised when generalizing the findings to left-behind children in other regions or cultural contexts. Future research should use cross-regional and multiethnic samples to further examine the cultural generalizability and contextual specificity of the proposed model.

Third, regarding the measurement of psychosocial adaptation, the single extracted factor explained 51.35% of the total variance, indicating that the six indicators shared a common psychosocial adaptation component. However, this also suggests that a substantial proportion of indicator-specific variance was not captured by the composite score. Therefore, the composite score is suitable for reflecting overall psychosocial adaptation, but it may not fully represent the specific characteristics of different adaptation indicators. Future research could further use methods such as latent profile analysis, combined with examinations of multiple specific adaptation indicators, to more finely explore the structure and related mechanisms of psychosocial adaptation.

Fourth, the internal consistency of the happiness scale was slightly low (α = 0.680), and the reliability of the depression scale was relatively limited (α = 0.724), which may have reduced measurement precision and attenuated the observed effect sizes. Although the sensitivity analyses using happiness and depression as separate outcomes supported the robustness of the main conclusions, future studies could use more reliable scales, alternative reliability indices such as McDonald’s ω, latent variable models, or multi-informant data to further validate the robustness of the multidimensional assessment of psychosocial adaptation.

Fifth, this study did not collect detailed information on parental migration patterns, such as father-only, mother-only migration, or both-parent migration. Therefore, we were unable to further compare whether the mechanisms underlying children’s psychosocial adaptation differed across different parental migration patterns. Different migration patterns may be associated with differences in caregiving arrangements, parent–child interaction frequency, emotional support, and family functioning, which may influence children’s stress mindset and psychosocial adaptation. In addition, the gender of the migrating parent(s) has also been shown to be an important factor influencing the psychosocial adaptation of left-behind children (Cheie and Prodan, 2025). Future research should systematically collect information on parental migration patterns, parental gender, and other relevant factors to examine differences in the mechanisms of psychosocial adaptation across different family contexts.

Sixth, this study primarily relied on self-report questionnaires, which may be subject to social desirability bias. Future research could incorporate multi-informant data, such as parent reports, teacher evaluations, or peer assessments, to improve the robustness and objectivity of the findings.

## 5 Conclusion

External assets were significantly and positively associated with psychosocial adaptation among left-behind children. Stress mindset mediated the association between external assets and psychosocial adaptation. Furthermore, environmental sensitivity significantly moderated the second stage of this mediation process. Specifically, the positive association between stress mindset and psychosocial adaptation was stronger among left-behind children with higher levels of environmental sensitivity. These findings provide preliminary cross-sectional evidence to inform future longitudinal and intervention research on the psychosocial adaptation of rural left-behind children.

## Data Availability

The raw data supporting the conclusions of this article will be made available by the authors, without undue reservation.
