# Supplementary material for: External assets and psychosocial adaptation in left-behind children: stress mindset as mediator and environmental sensitivity as moderator
Source: Front Psychol. 2026 Jul 2;17:1840516. doi: 10.3389/fpsyg.2026.1840516 (PMC13372585; doi:10.3389/fpsyg.2026.1840516)
Supplement: Supplementary file 1 [file Table_1.docx]

**Supplementary_Material_1**

To examine whether the findings based on the composite psychosocial adaptation score were robust when individual indicators were considered separately, sensitivity analyses were conducted focusing on depression and happiness, which represent the negative and positive dimensions of adaptation, and showed relatively low internal consistency.

As indicated in Supplementary Table S1, the results closely aligned with those obtained from the composite score. When depression was analyzed as the outcome, the interaction between external assets and environmental sensitivity was not significantly associated with depression (*β* = -0.003, *p* > 0.05). In contrast, the interaction between stress mindset and environmental sensitivity demonstrated a significant negative association with depression (*β* = -0.100, *p* < 0.001). The bias-corrected percentile bootstrap test showed that the index of moderated mediation was -0.028 (Boot *SE* = 0.006), with a 95% confidence interval of [-0.041, -0.016] that did not include zero. Similarly, when happiness was analyzed as the outcome, the interaction between external assets and environmental sensitivity was not significantly associated with happiness (*β* = 0.007, *p* > 0.05), whereas the interaction between stress mindset and environmental sensitivity was significantly and positively associated with happiness (*β* = 0.052, *p* < 0.01). The bias-corrected percentile bootstrap test indicated that the index of moderated mediation was 0.014 (Boot *SE* = 0.006), with a 95% confidence interval of [0.004, 0.027], excluding 0. Collectively, these findings indicate that the primary conclusions based on the composite psychosocial adaptation score remained robust when these representative positive and negative indicators were analyzed separately.

Supplementary Table S1. Sensitivity analyses of the moderated mediation model using depression and happiness as separate outcomes

| **Predictor variable** | **Model 1 (Depression as outcome)** | | | **Model 2 (Happiness as outcome)** | | |
| --- | --- | --- | --- | --- | --- | --- |
|  | ***β*** | **SE** | **95%CI** | ***β*** | **SE** | **95%CI** |
| Sex | 0.074 | 0.047 | [-0.018, 0.165] | -0.081 | 0.043 | [-0.165, 0.003] |
| Grade | 0.085 | 0.047 | [-0.007, 0.177] | -0.333*** | 0.043 | [-0.418, -0.248] |
| External assets(X) | -0.153*** | 0.024 | [-0.200, -0.105] | 0.288*** | 0.022 | [0.244, 0.332] |
| Stress mindset(M) | -0.244*** | 0.024 | [-0.291, -0.197] | 0.328*** | 0.022 | [0.285, 0.372] |
| Environmental sensitivity(W) | 0.199*** | 0.023 | [0.154, 0.245] | -0.024 | 0.021 | [-0.066, 0.018] |
| X×W | -0.003 | 0.022 | [-0.047, 0.041] | 0.007 | 0.021 | [-0.034, 0.047] |
| M×W | -0.100*** | 0.021 | [-0.141, -0.058] | 0.052** | 0.019 | [0.014, 0.089] |
| R² |  | 0.169 |  |  | 0.298 |  |
| F |  | 45.822*** |  |  | 96.120*** |  |
