# Supplementary material for: External assets and psychosocial adaptation in left-behind children: stress mindset as mediator and environmental sensitivity as moderator
Source: Front Psychol. 2026 Jul 2;17:1840516. doi: 10.3389/fpsyg.2026.1840516 (PMC13372585; doi:10.3389/fpsyg.2026.1840516)
Supplement: Supplementary file 2 [file Table_2.docx]

**Supplementary_Material_2**

Given the bifactor nature of the Highly Sensitive Child Scale-21 item version (HSC-21), supplementary factor-level moderated mediation analyses were conducted to systematically evaluate the role of its two specific factors-Ease of Excitation-Low Sensory Threshold (EOE-LST) and Aesthetic Sensitivity (AES)-as separate moderators in the proposed model. The empirical results, detailed in Supplementary Table S2, demonstrate highly consistent patterns with the primary findings based on the HSC-21 total score.

Supplementary Table S2. Moderated mediation analyses using environmental sensitivity dimensions

| **Moderator (W)** | **Predictor variable** | ***β*** | ***SE*** | ***t / z*** | ***p*** | **95% CI** |
| --- | --- | --- | --- | --- | --- | --- |
| EOE-LST | External assets (X × W) | -0.007 | 0.020 | -0.358 | 0.721 | [-0.047, 0.032] |
|  | Stress mindset (M × W) | 0.081 | 0.019 | 4.289 | < .001 | [0.044, 0.118] |
|  | *M-1SD* | 0.086 | 0.010 | — | — | [0.066, 0.106] |
|  | *Mean* | 0.108 | 0.011 | — | — | [0.087, 0.131] |
|  | *M+1SD* | 0.131 | 0.014 | — | — | [0.104, 0.159] |
|  | ZW_LST | 0.023 | 0.005 | — | — | [0.013, 0.033] |
| AES | External assets (X × W) | -0.012 | 0.020 | -0.569 | 0.569 | [-0.051, 0.028] |
|  | Stress mindset (M × W) | 0.063 | 0.019 | 3.245 | < 0.01 | [0.025, 0.101] |
|  | *M-1SD* | 0.091 | 0.011 | — | — | [0.072, 0.113] |
|  | *Mean* | 0.109 | 0.011 | — | — | [0.088, 0.132] |
|  | *M+1SD* | 0.126 | 0.014 | — | — | [0.100, 0.156] |
|  | ZW_AES | 0.018 | 0.006 | — | — | [0.006, 0.030] |

When EOE-LST was utilized as the specific moderator , the first-stage regression analysis revealed that the interaction between external assets and EOE-LST was not significantly associated with psychosocial adaptation (*β* = -0.007, *SE* = 0.020, *t* = -0.358, *p* = 0.721, 95% CI = [-0.047, 0.032]). Conversely, the second-stage regression analysis indicated that the interaction between stress mindset and EOE-LST exerted a significant positive effect on psychosocial adaptation (*β* = 0.081, *SE* = 0.019, *t* = 4.289, *p* < 0.001, 95% CI = [0.044, 0.118]). The bias-corrected percentile bootstrap test (with 5,000 resamples) further demonstrated that the conditional indirect effect of external assets on psychosocial adaptation via stress mindset systematically strengthened as EOE-LST levels increased: it was 0.086 at low levels (*M - 1SD*, Boot *SE* = 0.010, 95% CI = [0.066, 0.106]), 0.108 at mean levels (*Mean*, Boot *SE* = 0.011, 95% CI = [0.087, 0.131]), and reached 0.131 at high levels (*M + 1SD*, Boot *SE* = 0.014, 95% CI = [0.104, 0.159]). Crucially, the index of moderated mediation was statistically significant (*Index* = 0.023, Boot *SE* = 0.005, 95% CI = [0.013, 0.033]), confirming that EOE-LST successfully moderated the indirect pathway.

Similarly, when AES was implemented as the specific moderator , the interaction between external assets and AES was found to be non-significant (*β* = -0.012, *SE* = 0.020, *t* = -0.569, *p* = 0.569, 95% CI = [-0.051, 0.028]). In contrast, the interaction between stress mindset and AES was significantly and positively associated with psychosocial adaptation (*β* = 0.063, *SE* = 0.019, *t* = 3.245, *p* < 0.01, 95% CI = [0.025, 0.101]). The conditional indirect effect was statistically significant across all levels of the moderator: it was 0.091 at low AES (*M - 1SD*, Boot *SE* = 0.011, 95% CI = [0.072, 0.113]), 0.109 at mean AES (*Mean*, Boot *SE* = 0.011, 95% CI = [0.088, 0.132]), and peaked at 0.126 at high AES (*M + 1SD*, Boot *SE* = 0.014, 95% CI = [0.100, 0.156]). The index of moderated mediation confirmed the robustness of this conditional indirect pathway (*Index* = 0.0176, Boot *SE* = 0.0058, 95% CI = [0.0064, 0.0296]).

Collectively, these dimension-level sensitivity analyses rigorously reinforce the primary conclusions established by the composite score, corroborating that the conditional indirect effect of external assets on psychosocial adaptation via stress mindset is a robust phenomenon that holds universally consistent across both specific factors of environmental sensitivity.
